# Modularize and Unite: Toward Creating a Functional Artificial Cell

**DOI:** 10.3389/fmolb.2021.781986

**Published:** 2021-11-29

**Authors:** Chen Wang, Junzhu Yang, Yuan Lu

**Affiliations:** Key Laboratory of Industrial Biocatalysis, Department of Chemical Engineering, Ministry of Education, Tsinghua University, Beijing, China

**Keywords:** artificial cell, bottom-up construction, modularization, unity, function

## Abstract

An artificial cell is a simplified model of a living system, bringing breakthroughs into both basic life science and applied research. The bottom-up strategy instructs the construction of an artificial cell from nonliving materials, which could be complicated and interdisciplinary considering the inherent complexity of living cells. Although significant progress has been achieved in the past 2 decades, the area is still facing some problems, such as poor compatibility with complex bio-systems, instability, and low standardization of the construction method. In this review, we propose creating artificial cells through the integration of different functional modules. Furthermore, we divide the function requirements of an artificial cell into four essential parts (metabolism, energy supplement, proliferation, and communication) and discuss the present researches. Then we propose that the compartment and the reestablishment of the communication system would be essential for the reasonable integration of functional modules. Although enormous challenges remain, the modular construction would facilitate the simplification and standardization of an artificial cell toward a natural living system. This function-based strategy would also broaden the application of artificial cells and represent the steps of imitating and surpassing nature.

## 1 Introduction

With the million years of development, biological cells have established a high structural hierarchy and complex metabolic networks. The complexity has greatly hindered our understandings of the basic principles or mechanisms of life activities, probably limiting the future applications of life science. Moreover, scientists have always wondered whether a living cell can be artificially achieved or even synthesized *de novo*. Some synthetic compartments with cell sizes have attracted considerable attention as cell mimics, for their potential to uncover the inherent complexity of life. These are so-called artificial cells, which can help us to further explore the origin of life (Szostak et al., 2001; Rasmussen et al., 2004; Yang et al., 2013) and provide breakthroughs into application fields, such as drug delivery (Zhang et al., 2008; Liu et al., 2009; Ashley et al., 2011), gene therapy (Chang, 2005; Prakash and Jones, 2005), biosensing, and diagnostic (Pardee et al., 2016; Jayaraman et al., 2018).

Generally, there are two strategies to construct an artificial cell, top-down and bottom-up. The top-down strategy usually starts with a living cell. By eliminating or replacing the genome of a living cell, it can reach a “minimal cell” with minimal gene information for survival (Gibson et al., 2010). However, this strategy might be limited to the inherent complexity of living cells themselves. The other strategy, bottom-up, aims to construct a cell-mimic by assembling from nonliving materials. As Richard Feynman declared, “What I cannot create, I do not understand.” The bottom-up strategy can leave design space to customize artificial cells with unique properties, which indicates that it cannot only reconstruct a living cell, but also create an unnatural system to discover new possible life forms and develop new exciting applications.

The fundamental thinking to construct a bottom-up artificial cell can be summarized as the compartment of biochemistry networks, including gene replication, transcription, translation, metabolism, and signal transmission. The complicated reaction networks should be kept in a strictly organized and efficient state in a tiny space. Therefore, the compartment is a key process, as a cell mimic should not only be physically separated from the environment to form an independent individual but still keep in touch with the outside. Up to now, several materials have been applied to encapsulate the cell-free system. Lipids are most similar to the natural membrane, thus widely utilized to construct artificial cells (Noireaux and Libchaber, 2004; van Nies et al., 2018). Furthermore, other functional materials, such as polymers (Huang et al., 2013; Ugrinic et al., 2018), hydrogels (Park et al., 2009; Lai et al., 2019), and coacervates (Sokolova et al., 2013; Dora Tang et al., 2014), are also developed to meet new requirements. These compartments have provided a relatively stable interior and also enabled the communication between inside and outside (Shen et al., 2018; Lai et al., 2019).

As there is no clear and pervasive criterion for living, an artificial cell is usually synthesized to resemble one or more functions of a living cell and may differ from each other. Nonetheless, the ultimate goal is to reproduce cellular function and customize artificial cells based on personal application. The construction of bottom-up artificial cells has achieved significant progress since the 21st century. Simple biological functions can be realized inside a confined environment, including but not limited to the protein synthesis (Noireaux et al., 2005; Park et al., 2009), photosynthesis (Lee et al., 2018; Berhanu et al., 2019), membrane proliferating and division (Matsuo et al., 2019), and signal transmission (Niederholtmeyer et al., 2018; Joesaar et al., 2019).

**FIGURE 1:**
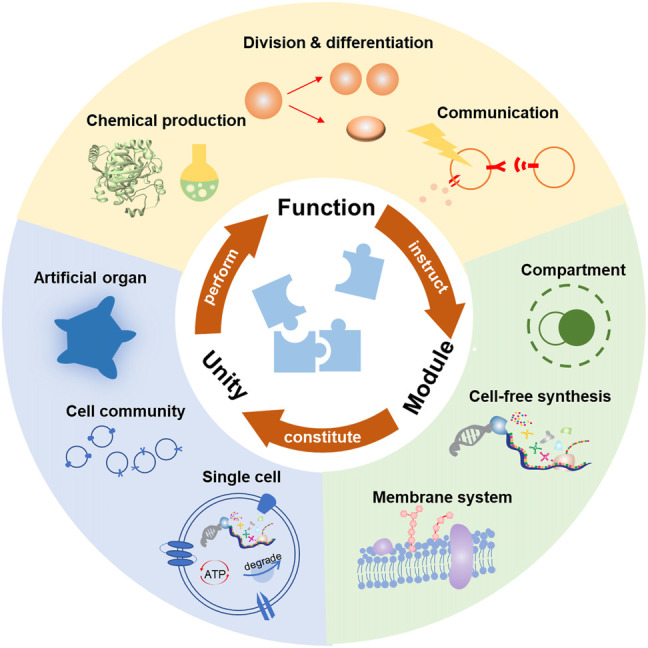
Bottom-up construction of artificial cells based on the functions. Considering the inherent complexity of living cells, constructing artificial cells through various functional modules seem feasible. The effective assembly of these modules can finally perform specific functions, such as chemical production, division and differentiation, or communication. Conversely, the desired functions can instruct the creation of various new modules. In this case, the customization of artificial cells based on the desired functions might eventually be realized.

However, as natural cells are quite different from each other and capable of diverse life activities, it would be impossible to mimic every specific function. An effective strategy is to rebuild the functional networks by combining several basic blocks, just like Lego. In this case, people could create what they need through a standardized method. From the common ground of natural cells, the basic functions could be summarized into four aspects: substance metabolism, energy supplement, proliferation, and communication. Generally, the substance metabolism promotes dynamic communication among substrates, thus providing the material base for life. As most life activities are energy dissipative, it is crucial for the artificial cell to gain sufficient energy to support its functions. Another specific characteristic of a living system is the ability of proliferation, that is, to grow, copy themselves, and split. There seem to be three distinctive processes, but they should be considered as a whole for their equally critical efforts toward the subsequent generations. Moreover, an artificial cell should also have the ability to exchange information with and respond to the environment or other living systems. Based on the communication, it may adjust the internal activities, which paves the way for the adaptive evolution toward a real living system (Szostak et al., 2001). In the following context, we mainly focus on the latest development of these function modules, the basic principles of construction, and their development in the future. Then we discuss their combination toward a complex artificial cell. Here we demonstrate that the unity should observe several disciplines to realize efficient synergistic behaviors. Through the integration of these four modules, one can realize the full complexity of a living cell in a methodical way or even customize the artificial cell with unnatural functions (Figure 1).

## 2 Modularization

### 2.1 Substance Metabolism

**FIGURE 2:**
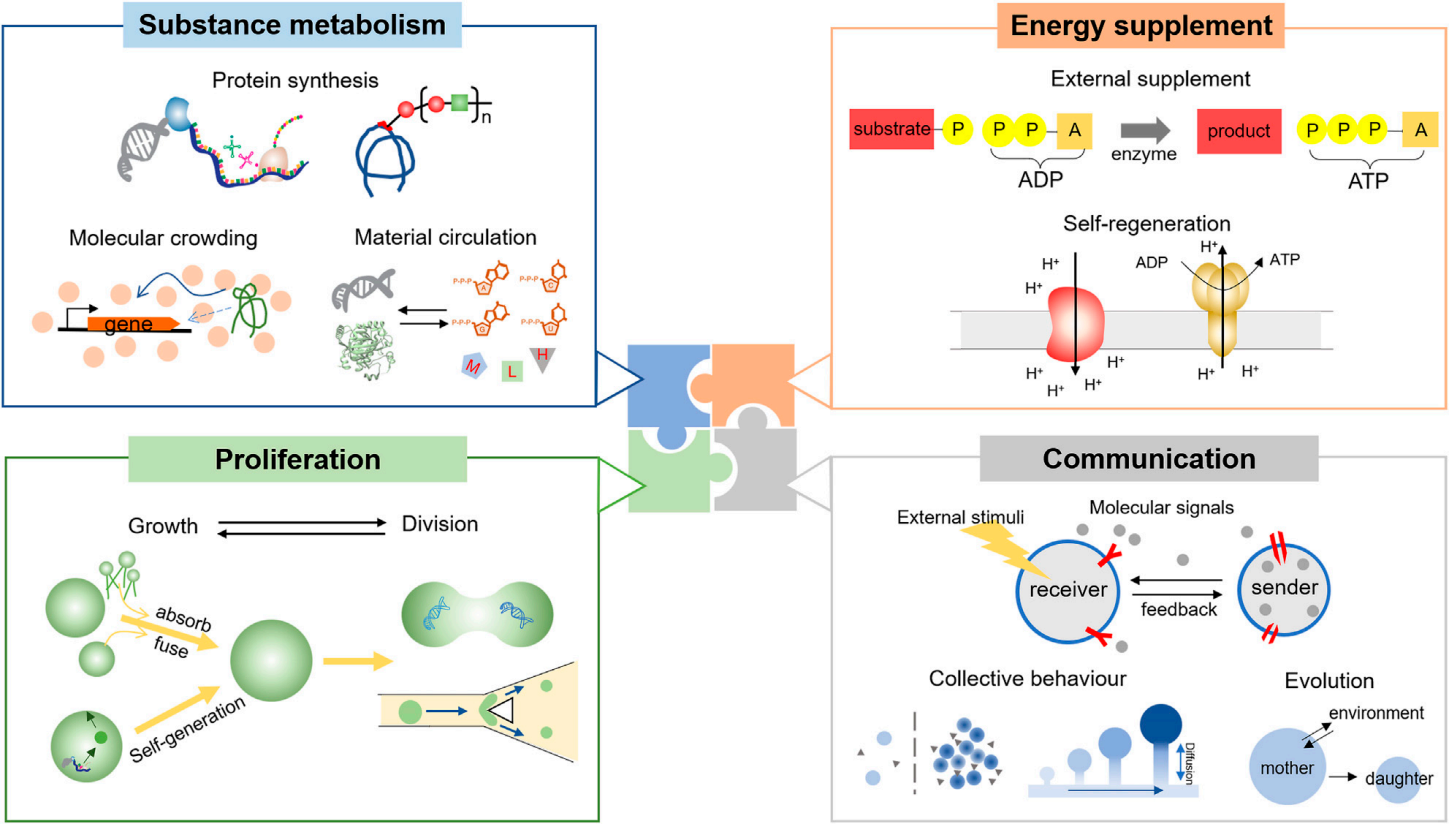
The function of artificial cells could be divided into four parts: substance metabolism, energy supplement, proliferation, and communication. Since proteins play a great role in cellular behaviors, the protein synthesis is the basic function of artificial cells. To establish an efficient and sustainable substance metabolism, the imitation of the crowded intracellular environment is crucial, as well as the construction of the material circulation. Energy supplement is also important, considering that most life processes are energy-dissipative. Currently, the supplement methods are classified as the external supplement or the self-regeneration model. The latter one is more advantageous toward creating living cell-mimics. The proliferation of an artificial cell could be split into the growth and division processes. However, the accurate allocation of the interior remains difficult. Finally, an artificial cell should have the ability to communicate with the environment and each other. Collective behavior should be considered in cell-mimic communities.

Metabolism describes the overall biochemical reactions in a living organism, which meets the essential material requirement for survival and provides conditions for advanced functions, such as communication. In this case, the construction of metabolism inside an artificial cell is significant and endows the tiny compartment with various functions as a living cell. Here we mainly focus on the substance conversion that happens in biological processes, such as the synthesis of macromolecules, and argue how to construct effective substance metabolism in an artificial cell (Figure 2, Substance metabolism).

#### 2.1.1 Cell-free Protein Synthesis System

Synthesizing proteins is crucial for living cells, as these biomacromolecules take a significant role in almost all cellular behaviors. They have complicated quaternary structures and can function as structural supports of organisms or various bio-catalysts. Therefore, producing proteins on-demand in an independent and efficient way is fundamental for a biomimetic artificial cell. A cell-free protein synthesis (CFPS) system (Nirenberg and Matthaei, 1961) is an *in vitro* system, which enables transcription and translation processes outside cells. It simplifies the complex interactions in a living cell and only consists of the necessary elements for protein synthesis, such as gene templates, related enzymes, amino acids, salts, energy substrates, and cofactors. The principle behind CFPS is the biological central dogma, which refers to the transmission of genetic information from DNA to message RNA, and finally toward proteins. Due to the openness, simplicity, and operability, the CFPS system can provide an ideal platform for protein synthesis in artificial cells (Shin and Noireaux, 2012; Wu and Tan, 2014). Moreover, protein synthesis using recombinant elements (PURE) (Shimizu et al., 2001; Lavickova and Maerkl, 2019) system could completely customize the artificial cells, as its components are entirely purified and remain minimal but necessary for protein synthesis. For example, by adjusting substrates and enzymes in the PURE system, the metabolic pathways were controlled, and the detailed roles of ATPs were revealed in an ATP-producing artificial cell (Berhanu et al., 2019).

Although successfully producing model proteins like fluorescent proteins (Shin and Noireaux, 2012; Caschera and Noireaux, 2014), CFPS systems should be furtherly expanded to support the synthesis of complex and specific proteins. For example, membrane proteins are common proteins that perform crucial functions for cell survival (Almén et al., 2009). They could serve as enzymes, signal receptors, and transport machines. Therefore, the synthesis of membrane proteins has connected the boundary and the interior, thus facilitating the construction of artificial cells as an organic whole. Cell-free systems could provide an attractive platform to produce membrane proteins by adding surfactants or liposomes (Lu, 2017). These methods might still function in a membrane-bounded artificial cell. Several membrane proteins have been successfully synthesized and functioned in artificial cells, such as bacteriorhodopsins (Berhanu et al., 2019), cell division proteins FtsA (Furusato et al., 2018; Godino et al., 2020), and *E. coli* Min proteins (Godino et al., 2019). However, the correct assembling of *de novo* synthesized membrane proteins remains inefficient in artificial cells, partly due to the unclear integration mechanism of proteins into bio-membranes.

Another challenge is the post-translation modification of proteins *in vitro*. Most proteins require further modification after the translation process, such as glycosylation and phosphorylation, to become mature and undertake advanced physiological functions (Wang Y.-C. et al., 2014). However, these might be difficult or inefficient *in vitro*, as the explicit modification processes are often complicated, involving multiple metabolites (Kightlinger et al., 2019). Some countermeasures are the fabrication of cell extracts in CFPS from eukaryotic cells and the supplement of disulfide bond-forming enzymes or chaperones (Goerke and Swartz, 2008; Lu, 2017). Up to now, results have been achieved to introduce the complete enzyme systems or pathways for post-translation modifications in the cell-free solution (Kightlinger et al., 2019). Nevertheless, the ability of artificial cells to support modification processes needs to be improved, as they would be crucial in the construction of diverse metabolic pathways and other advanced cellular functions.

#### 2.1.2 Imitation of Intracellular Environment

Intracellular homeostasis is important for normal metabolic activities. A significant difference between cells and solutions is the crowded environment inside the former. About 30% of the volume is occupied by macromolecules such as proteins, nucleic acids, and polysaccharides, whose concentration can reach 300–400 g/L (Zhu et al., 2020), and even 500 g/L in some compartments inside cells (Minton, 2015). The high concentration of macromolecules causes the so-called “macromolecular crowding” and brings significant effects to the interactions between participating biomolecules and the final metabolism (Rivas and Minton, 2016). First, the crowding environment can cause the excluded volume effect, which reduces the diffusion coefficients of both small and large molecules (Tan et al., 2013; Smith et al., 2017). Second, it also increases the equilibrium constants associated with macromolecules, probably due to the decrease in the total free energy of the solution after binding to molecules (Ellis, 2001). Moreover, the crowding effect can enhance the association of proteins, especially facilitating the folding or aggregation of proteins (Ellis, 2001; Bai et al., 2017). As bioreactions could be affected by both the diffusion process and molecule activities, the crowding effect on distinct bioreactions could be complicated with the increased concentration of macromolecules. In this case, creating a similar physiological medium inside an artificial cell deserves great attention.

Although the direct encapsulation could form a restricted interior, the concentration of macromolecules is usually not able to reach as high as in living cells (Minton, 2006). An intuitive strategy is to add inert crowding agents, such as poly (ethylene glycol) (PEG), dextran, and ficoll (van den Berg et al., 2017). These generally drift away from the major metabolism and might perform differently according to their species and concentrations (Ge et al., 2011). Therefore, this method might be limited to some extent. Considering the mismatching of diluted cell-free system with the large size of compartments, it would also be applicable to condense the cell-free reaction mixtures (Fujiwara and Nomura, 2013) and adjust the size of artificial cells through osmotic pressure regulation to create a more crowding internal environment. For example, monodisperse liposomes could shrink three orders of magnitudes to form crowded artificial cells (Deng et al., 2018). The interior solution finally reached about 272 g/L, which was close to the living cell.

Reconstitution of the crowded environment in an artificial cell is crucial in a broader sense. It causes the cytoplasm to be more similar to gels rather than solutions, and even leads to the lipid-lipid phase separation (LLPS) to get membrane-free compartments (Ma et al., 2020). These intracellular structures are involved in diverse metabolism in both eukaryotes and prokaryotes (Wei et al., 2020), and have been proved to benefit the protein synthesis (Sokolova et al., 2013; Tang et al., 2015; Deng et al., 2018). Condensates inside artificial cells were successfully triggered through LLPS to conduct metabolic behaviors, such as enzyme catalysis (Deshpande et al., 2019a; Love et al., 2020), and study the potential coacervate-membrane interactions (Last et al., 2020). In a complicated metabolic network, these membrane-less organelles would ensure orderly biochemical reactions and provide valuable insights toward the real metabolism inside cells (Deshpande and Dekker, 2021). In this case, the crowded internal homeostasis and the local confinement would help construct an efficient and robust metabolic network inside an artificial cell.

#### 2.1.3 Complex Metabolism in Artificial Cells

The encapsulation of one single enzymatic reaction would be relatively simple in the artificial cell. However, the complex bioreaction cascades or networks seem challenging because of the complicated processes and ambiguous mechanisms. Here, we propose that the recycling processes, which mainly refer to the circulation of metabolites and enzymes, should be carefully considered to construct complicated metabolism. In this case, more steady output and complex metabolic operation could be realized.

A robust and sustainable reaction network relies on stable conditions, which could be simplified as the continuous supply of substrates and elimination of products. This was primarily raised to prolong the life of cell-free protein synthesis, mainly through the selective permeability (Spirin et al., 1988) or anchor of the pore proteins onto membranes (Noireaux and Libchaber, 2004). After that, nanoliter-scale reactors, which could constantly exchange all molecules, were constructed to carry out diverse regulatory mechanisms (Niederholtmeyer et al., 2013). Meanwhile, the auto-regulated protein synthesis was achieved based on the on-chip DNA compartments (Karzbrun et al., 2014). These results proved the necessity of steady-state conditions for complex and continuous metabolic regulation. Recently, hydrogels with porous structures were applied to encapsulate the PURE system and support genetic oscillators through the continuous exchange of substrates and byproducts (Zhou et al., 2018; Lai et al., 2019). The circulation of materials could provide an opportunity for the construction and regulation of complicated metabolic networks.

Another crucial factor is the regeneration and digestion of biomolecules, such as nucleic acids and enzymes. As these biomolecules could lose activity during the reaction process, it is important for sustainable artificial cells to break them down and synthesize new substitutes. However, the *de novo* synthesis of original components is not a trivial task. On the one hand, most complex biomolecules are still hardly produced for the low efficiency or complicated assembly *in vitro*. On the other hand, as some functional enzymes prefer to function on membranes, the correct location is also essential. Up to now, significant progress has been made in reconstituting several vital biomolecules evolved in metabolism, including DNAs (van Nies et al., 2018; Libicher et al., 2020), ribosomes (Jewett et al., 2013; Shimojo et al., 2020), and tRNAs (Hibi et al., 2020). Recently, a partial self-regeneration artificial cell system capable of regenerating essential components was also constructed successfully (Lavickova et al., 2020). These results were mainly based on the minimal reaction demand. The next step would be substrate recycling in more complicated metabolic networks.

As for the digestion part, hydrolytic enzymes are commonly used in living cells for the digestion of biomolecules. However, their optimal pH is often at 4.5–5. To provide a proper degradation environment and prevent interference with other bioreactions, the encapsulation of hydrolytic enzymes as a lysosome-like module seems applicable. Actually, lysosomes inside eukaryotic cells consist of many hydrolytic enzymes, and the guidance of inactive biomolecules toward digestion is complicated and still under exploration (Bonam et al., 2019). Carreira et al. (2017) developed a type of lysosome-mimicking vesicles and discovered that the accumulation of sphingosines could cause the permeability and other biophysical changes of the membrane, which might facilitate the digestive function of lysosomes. The degradation of enzymes or other biomolecules is generally a necessary part of metabolism. Conclusively, more efforts should be made to construct an efficient recycling system inside an artificial cell or between artificial cells and the environment.

### 2.2 Energy Supplement

To maintain the complicated and sustainable metabolism as well as the homeostasis, an artificial cell should be supplied with sufficient energy. In a living cell, ATPs are the most common energetic substrate, whose generation and consumption are coupled with various bioreactions. Generally, there are three methods to produce ATPs in nature, including substrate-level phosphorylation, photo-phosphorylation, and oxidation-phosphorylation. Inspired by these three processes, different strategies have been adopted to provide ATPs in a synthetic cellular system (Figure 2, Energy supplement).

#### 2.2.1 External Supplement

The substrate-level phosphorylation refers to the direct coupling of energy-rich compounds with ATP production. Phosphate groups are extracted from the substrates and transferred to ADP or GDP to produce ATP or GTP. Because of the simplicity and easy-operating, this is widely applied in cell-free systems. Traditional high-energy substrates include phosphoenolpyruvate, creatine phosphate, and acetyl phosphate, which could support protein synthesis at the level of g/L (Des Soye et al., 2018). However, the phosphate compounds could hamper the synthesis in a batch reaction, for their high costs, the accumulation of inorganic phosphate, and the pH change (Jewett and Swartz, 2004; Kim et al., 2007). In this case, glycolytic intermediates such as pyruvate (Jewett and Swartz, 2004), maltodextrin (Kim et al., 2011), and maltose (Caschera and Noireaux, 2014) were introduced for the recycling of inorganic phosphate, thus facilitating the ATP regeneration and improving the homeostasis of the cell-free system. Nonetheless, they might suffer from the inefficient utilization of the ATPs and other unexpected byproducts, as additional metabolic pathways were generally included (Caschera and Noireaux, 2014).

Another approach is to build a material exchange system. Through the continuous supply of energy and removal of harmful byproducts, artificial cells could reach a longer lifespan. For example, dialysis membranes were used to separate the cell-free system from the feeding buffer (Kim and Choi, 1996). Therefore, small molecules such as the energy substrates could diffuse across the membrane and support sustainable protein synthesis. Furthermore, pore-forming proteins were also anchored onto the membrane to improve the mass transfer (Noireaux and Libchaber, 2004). Recently, artificial cells based on porous hydrogels were constructed to conduct long-lived protein synthesis (Zhou et al., 2018; Lai et al., 2019). Although the interaction with the environment was a primary characteristic of a living system, the continuous supply of energy substrates might be limited in practice by the high costs and the specific feeding system.

#### 2.2.2 Self-Regeneration System

It is worthwhile to construct a self-regeneration system for energy in an artificial cell from the perspective of reconstructing life. In nature, living cells can produce ATPs efficiently through the oxidation-phosphorylation and photo-phosphorylation processes, which can be summarized in two steps (Jeong et al., 2020). First, a proton gradient is established across the membrane boundary. In the oxidation-phosphorylation process, the driving force can be attributed to the oxidation of energy-rich molecules, in which electrons are transported through the electron transport chains (ETCs) (Nath and Villadsen, 2015). While in the photo-phosphorylation, it is driven by light with the help of photosynthetic pigments. Second, the proton gradient drives the ATP synthase (Nesci et al., 2015) to produce ATPs. The recovery of these two processes inside an artificial cell might lead to a self-sufficient supply of energy.

The construction of the ATP synthesis system can date back to 1974, when the purple membrane from *Halobacferium halobium* was packaged into phospholipid vesicles to support light-driven ATP synthesis (Racker and Stoeckenius, 1974). After that, the reconstitution of purified bacteriorhodopsin (bR) and ATP synthase in proteoliposomes enabled the formation of the proton gradient and ATP synthase (Sone et al., 1977). This indicated the possibility of ATP synthesis from completely synthetic components. In the next 30 years, the efficiency and stability of the ATP synthesis system were improved through the reconstitution of ATP synthases from bacteria (Richard et al., 1995; Choi et al., 2006), plants (Feng et al., 2016; Li et al., 2019) or mitochondria (Matuschka et al., 1995), and different types of proton gradient generators (Li et al., 2019; Tarryn et al., 2020; Xu et al., 2021). However, the membrane materials were generally based on lipids, probably due to their excellent compatibility with membrane proteins. Therefore, the various lipid compositions were studied to test their effects, and the protein densities were found to play a great role in ATP synthesis (Nilsson et al., 2016). In this case, the ATP synthesis system, or namely the artificial organelle for energy generation, was established in an efficient way.

These pre-prepared modules could be integrated into vesicles to drive other bioreactions. In 2018, a switchable ATP synthesis system, which could respond to light with different wavelengths, was coupled with actin polymerization and finally led to membrane growth (Lee et al., 2018). Then, in the next year, a similar photosynthetic organelle composed of bR proteins and ATP synthases was introduced into the recombinant cell-free system to support the complex transcription and translation processes (Berhanu et al., 2019). This fascinating result proved the compatibility of self-sufficient ATP synthesis with protein production. Recently, a chloroplast mimic with natural and synthetic parts was constructed to conduct a complete metabolic cycle for continuous CO_2_ fixing (Tarryn et al., 2020). Although significant progress has been achieved, these synthetic energy modules might still suffer from low efficiency. Most of them were only utilized in simple reaction cascades. A possible solution might be the loading of more functional proteins. For instance, the vast surfaces of mitochondria and chloroplasts in nature have enabled the complex metabolic networks inside cells. Therefore, the construction of membrane structures with a considerable surface area could facilitate the energy supplement in logic and finally help construct an energetically independent artificial cell.

### 2.3 Proliferation

Proliferation is a typical feature of a living cell, which generally refers to the generation of two daughter cells from one mother cell. This facilitates the population expansion of unicellular organisms and the development of advanced species. Moreover, the correct and steady proliferation has provided foundations for the Darwinian evolution, as the offspring should inherit most of the characteristics and a few mutations from parent generations. The detailed processes of proliferation have not been completely revealed. In this case, the construction of reproducible artificial cells could help us understand the possible mechanisms, and may play a role in synthetic tissues and organs, or other applicable fields. For an accurate description, here we mainly discuss the growth and division of one single artificial cell (Figure 2, Proliferation).

#### 2.3.1 Growth

Growth represents the volume increase and mainly happens prior to the division. The metabolism should also get enhanced in this process. Generally, artificial cells could grow in three forms: absorbing materials from the environment, fusing with each other, and creating the materials from inside.

Membrane vesicles composed of fatty acids could absorb alkaline micelles spontaneously from outside and were first studied to imitate the cell growing process (Chen and Szostak, 2004; Stano and Luisi, 2010). This model might illustrate the state of primitive cells, but was probably not suitable for artificial cells with complex functions, as fatty acids were not compatible with some biological reactions and limited as a membrane material (Schwille et al., 2018). Afterward, phospholipid-based vesicles were successfully constructed to grow by fusing with each other. Giant unilamellar vesicles (GUVs) could gradually grow when supplied with small unilamellar vesicles (SUVs). The processes were mediated by the membrane tension through a transmembrane osmotic pressure (Deshpande et al., 2019b). To avoid the disordered fusion, the lipid membranes could be modified by specific molecules so that the fusion process could be initiated in the desired manner. For example, by using negatively charged lipids, the fusion of vesicles could be achieved through electrostatic repulsion (Terasawa et al., 2012). The growing process could also be light-controlled through the use of photo-sensitive surfactants (Suzuki et al., 2017). Besides, the protein-lipid interaction was proved promising for the induction of membrane tethering and fusion (Cai et al., 2019). Some polymer-based vesicles could grow upon integrating stimuli-responsive nanoparticles, which contained the membrane materials (Schwille et al., 2018). Compared with the absorption mechanism, the fusion process could supply other necessary substrates to meet the metabolic burden brought by the volume increase, thus might be popular in future studies. These two strategies both rely on the acquisition of the homologous membrane materials from the environment and reflect a primary growing form. A more lucrative way is that artificial cells synthesize their own membrane components and integrate them on the preexisting membrane. The synthesis of phospholipids has been achieved by purified enzymes *in vitro* (Schmidli et al., 1991), which provided a solid precondition. Recently, membrane growing was realized through the internal biosynthesis of lipids (Exterkate et al., 2018; Blanken et al., 2020). These excellent works would greatly improve the construction of an autonomous and self-reproducible artificial cell. Except for the volume increase, the morphological change was another growing form, such as the axon outgrowth. Artificial cells growing into asymmetric forms might help us to understand cell differentiation and deserve more exploring.

#### 2.3.2 Division

As the next step of growth, division is almost inevitable for most cells. In this process, the cell membrane would deform to decrease its volume-to-surface ratio and become the dumbbell shape connected by a narrow membrane neck. Meanwhile, the interior is distributed into two different compartments. After the collapse of the membrane neck, the interior is separated, and the cell eventually divides into two daughter cells. Inspired by this, the division of an artificial cell could be resolved into the division of compartments and the allocation of crucial components such as genetic materials and enzymes. In the following text, these two aspects will be discussed in details.

GUVs are broadly used to study the division process, as they possess great fluidity and are most similar to natural membrane systems. By constructing fissionable GUVs, several models of membrane division induced by physical mechanism, chemical synthesis, or fundamental biological processes had been established (Caspi and Dekker, 2014). For example, some division machinery based on structure proteins, such as actomyosin and FtsZ, were found necessary in the division cycle. After the reconstitution of these proteins on GUVs, the liposome division could be observed in some cases (Osawa and Erickson, 2013). Besides, the cell-free synthesized bacterial division proto-rings could help to constrict liposomes and generate budding vesicles (Godino et al., 2020). This model relied on the specific splitting proteins anchored on lipid membranes. However, membrane fission could also be induced regardless of the protein structure. The steric pressure among the closely arranged membrane proteins could increase the membrane curvature and finally lead to a division. As a result, even green fluorescent proteins bounded on the membrane could drive fission efficiently (Snead et al., 2017). Furthermore, even low-density membrane proteins were feasible to induce the vesicle division by the precise control of the spontaneous membrane curvature (Steinkuhler et al., 2020). These efforts greatly decrease the complexity of the division process *in vitro*. However, they often resulted in unpredictable division results both of the volume and the components. As a result, microfluidics was applied to cut liposomes mechanically, thus precisely controlling the generation of daughter cells (Deshpande et al., 2018). This strategy could produce homogeneous offspring but be limited by the size of the vesicles and the specific wedge-shaped splitter. Recently, the spatiotemporal control of division was realized through the suitable osmolarity change based on the phase-separated GUVs. In this case, only the target GUVs would divide, while the others were not affected (Dreher et al., 2021). Based on these current works, one typical issue is that the allocation of the volume and components seems pretty random during the membrane fission. Considering the complex metabolism encapsulated inside vesicles, the precise and average division of the artificial mother cell would be pretty difficult. Some breakthroughs were achieved by the coupling of compartments and gene materials. Through the interaction between the negatively charged DNA and the cationic membrane, the division of the compartments could be linked with the amplification of DNA (Kurihara et al., 2011). As a result, the latter could significantly induce the growth and division of vesicles. On the other hand, RNA as another gene information carrier was encapsulated inside fatty acid vesicles and successfully distributed to the daughter cells (Zhu and Szostak, 2009). Nevertheless, these models could not illustrate the precisely average distribution of maternal genetic information. This is a crucial question, as the volume and other components might be separated mechanically through microfluidics, but the information materials should be replicated and distributed accurately.

Although the growth and division of artificial cells have distinctive processes, they all experience the change in volume or morphology. Especially, the asymmetric division, in which the offspring can inherit different components, should not be neglected, as it is inevitable in cell differentiation and various life course (Andes-Koback and Keating, 2011). In reference to the natural processes, the accurate distribution of gene materials and the precise control of division, both average and asymmetric, would be the focus of future works.

### 2.4 Communication

The three function modules discussed above were mainly based on one single artificial cell. However, information exchange is also a fundamental characteristic of a living cell, which means that it should react to various stimuli from the environment and cooperate or confront each other. Communications also broadly happen inside the compartments among interrelated function modules, as feedback is necessary to regulate complicated reaction networks. Here we would mainly talk about the communication between artificial cells and the environment, or among artificial cells (Figure 2, Communication).

#### 2.4.1 Basic Models

Generally, the communication process requires three elements, the sender, the receiver, and the signal-transmitting information. The senders catch the signals released by the receivers and make responses accordingly. The signals could be physically, chemically, or biologically originated. The response could be the production or release of target molecules, cell motility, or just the simple changes in morphology. The simplest model of the artificial communication process is that an artificial cell receives signals from the environment and takes specific responses. For example, semipermeable compartments could allow the exchange of reaction substrates, thus facilitating protein synthesis (Zhou et al., 2018; Lai et al., 2019). The intelligent release of agents was realized based on the induction of various environmental stimuli, including pH, osmotic pressure, light, ultrasound, and temperature (Fomina et al., 2010; Wang Y. C. et al., 2014; Basuki et al., 2017). However, the signal sources are not rigidly limited, which can also be artificially supplied. A more complex communication model requires specific signal senders. In this case, scientists aimed to construct another group of artificial sender cells, which released signals to the environment (Adamala et al., 2017; Joesaar et al., 2019). Except for all syntheses, natural cells could also be used as senders or receivers to interfere with artificial cells (Elani, 2021). Furthermore, based on the sender-receiver model, feedbacks could be introduced rather than one-way communication. For example, while the senders were the receivers themselves, they could regulate bioreactions based on their own products, which showed interesting collective behaviors as a community (Gines et al., 2017). Besides, the bidirectional communication was realized between artificial cells based on DNA strand-displacement circuits (Joesaar et al., 2019). Nevertheless, the typical communication types are not so rich. The construction of multi-directional communication still needs further studies, as well as the precise control of information processing.

#### 2.4.2 Simple Principles

Signals hold an essential place in an artificial communication system, for they relate to the design of artificial cells and determine the transmission type. As discussed in Section 2.4.1, signals could be various. The intuitive method of receiving external signals utilizes stimuli-sensitive materials to construct receivers (Wang Y.-C. et al., 2014). These materials could change their morphology, swell or shrink, or modify their pore structures to regulate the transmission of signal molecules (Yavuz et al., 2009; Yan et al., 2013; Wang et al., 2020). More generally, it is applicable to introduce controlling modules from gene level, such as light-activated T7 promoter (Booth et al., 2016), various biosensors for small molecules, or specific ions (Jung et al., 2020; Thavarajah et al., 2020), to regulate the gene expression. This strategy is not limited by the sensitive materials. After the integration of different sensing modules inside one compartment, more complicated communication abilities would be practicable in artificial cells.

Another remarkable point is the transmission style of signals if information molecules are applied. Artificial cells could release or receive signals just through the diffusion of chemical molecules. Compared with the macromolecules, smaller molecules like CO_2_ (Yan et al., 2013), glucose (Arya et al., 2016), N-(3-oxo-hexanoyl)-L-homoserine lactone (3OC6HSL) (Lentini et al., 2017), can pass through the semipermeable membranes more easily, thus are broadly used as signals. Large molecules are also applicable, but they generally need large pore sizes or just membrane-free compartments. For example, DNA segments could diffuse into proteinosomes with porous structures to initiate the protein synthesis (Joesaar et al., 2019). Artificial cells based on clay hydrogels possessed large pores and even communicated through diffusive protein signals (Niederholtmeyer et al., 2018). These porous materials were more tolerant for molecular transfer than the traditional liposomes. Nonetheless, it would be more difficult to modify their surface with functional biological macromolecules compared with liposomes (Rideau et al., 2018). The impermeable molecules could go through the lipid membrane by the reconstitution of membrane pores, such as α-hemolysin (Adamala et al., 2017; Hilburger et al., 2019). These transmission processes were mostly driven by passive molecular motion and almost uncontrollable. They might be inefficient and interfere with each other in a complicated system with multi signals. To solve this problem, communications based on specific interactions have provided a feasible idea. For instance, the antibody binding could selectively target the specific bacterial and trigger a response when supplied in two groups of bacterial (Fernandes et al., 2010). Similar interaction includes the avidin-biotin complexation, which was realized in a synthetic vesicle-to-vesicle communication system (Ding et al., 2019). These specific interactions mostly rely on the biomolecules anchoring on the membrane, and thus the bio-compatibility and modifiability of membrane materials are extremely important.

#### 2.4.3 Various Collective Behaviors

Communication is a kind of group behavior, which involves a large number of individual artificial cells. Based on the sender-signal-receiver models mentioned above, various forms of communications could be established, whether they exist in reality or not. Quorum sensing (QS) is pretty common in bacterial populations, which refers to the regulation of gene expression in response to the concentration of autoinducers produced by bacteria themselves (Miller and Bassler, 2001). Quorum sensing is usually determined by the cell-population density. For instance, fluorescence could accumulate in artificial cells only at high densities by using T3 RNA polymerases as QS signals (Niederholtmeyer et al., 2018). The signal molecules could also spread and form spatial distribution to induce different activities like pulses and waves in the artificial cell groups (Tayar et al., 2015). In contrast to this heterogeneous phenomena, synchrony and pattern formation were also realized through the nonlinear genetic oscillators coupling on a chip of artificial cells (Tayar et al., 2017). However, this collective behavior was still hard to be reproduced *in vitro*, probably due to the low robustness of cell-free oscillators. Another exciting application of the synthetic communication process is to mimic the natural differentiation of cell groups, which is the basis of development for advanced species. For example, by implementing different types of feedback gene circuits, artificial cells could exhibit differentiated gene expression during the signaling process (Dupin and Simmel, 2019). In a word, the studies of synthetic communication systems would not only improve our cognitions of collective cellular behaviors but facilitate the construction of a dynamically connected artificial cell community.

#### 2.4.4 Evolution

Evolution is a typical and crucial characteristic of life (Szostak et al., 2001; Schwille et al., 2018). Here we put it in this section as it reflects the communication between artificial cells and the environment. The artificial cells can react to the environmental pressure and take a response to change themselves, which could be inherited by their future generations. Therefore, the evolution process is largely related to the information coupled with the fitness of artificial cells. A typical model was that RNAs with special structures coupled with the interior vesicle wall could facilitate the stability of vesicle and improve survival (Szostak et al., 2001). The complementary pairing between short oligonucleotides and replicase ribozymes was also studied as an evolution system model (Engelhart et al., 2016). The oligonucleotides could combine with the ribozymes and inhibit vesicle growth. In addition to the typical response-receive model, the tight connection between the response and the survival of artificial cells is also required to provide the evolution engine. Since the proliferation of artificial cells has not been stably achieved, the construction of artificial evolution systems still has a long way to go. However, it should be considered necessary to build a self-sustaining artificial cell.

## 3 Unity

**FIGURE 3:**
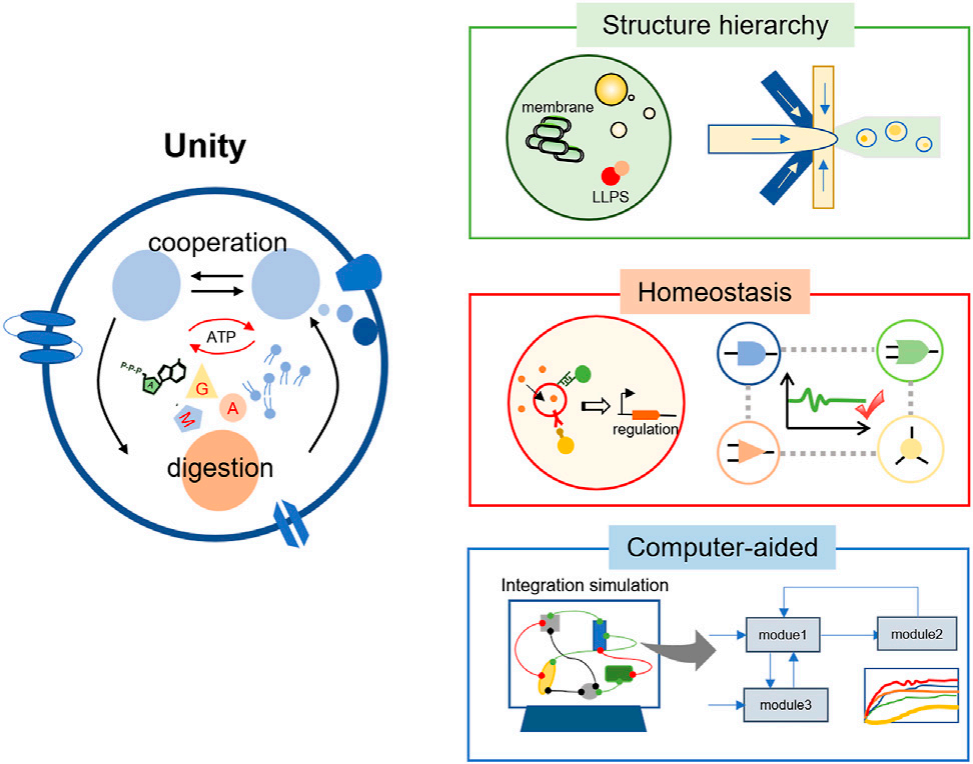
The integration of different modules into a functional artificial cell. Reestablishing the structure hierarchy of living cells is practical, which refers to the multicompartment and the complex membrane system inside an artificial cell. In addition, the regulation system of different modules should be constructed to maintain the homeostasis. Considering the extremely complicated interactions, the computer-aided technology would have wide applications in the integration of basic models and even the design of a complete artificial cell.

The increased library size and quality of building blocks would broaden the diversity and functionality of artificial cells. The next question is how to assemble these modules into a complete artificial cell (Figure 3). This idea could be naturally generated. For example, when constructing a light-powered or communicative artificial cell, the intuitive evaluating method is to couple the target process with the synthesis of fluorescent proteins. However, with the increasing amounts of building blocks, they might be interpreted by each other and counteract to decrease the efficiency as a whole. A typical example could be the competition between different modules for the common substrates.

Millions of years of evolution have endowed living cells with a complex structure to overcome this difficulty. A natural eukaryotic cell has various organelles with different compositions and assumes different cellular functions (Zhu et al., 2020). Even prokaryotic cells have nucleoids and ribosomes. This hierarchy helps to separate different modules to ensure they function in a proper condition. The coordination of various organelles has made cells a highly-ordered biochemical machine. This phenomenon inspired us that the multicompartment structure could help to integrate the building blocks toward a complicated artificial cell. Through encapsulating functional modules into different sub-compartments, one can achieve the spatiotemporal organization inside an artificial cell. Simple two-dimensional nesting structures could be formed through careful regulation during the fabricating of large vesicles (Dora Tang et al., 2014; Booth et al., 2019). However, this method could be primarily influenced by the operators when dealing with more complex architecture. On the other hand, the nested droplets produced by microfluidics have been studied as mimics of eukaryotic cells (Ai et al., 2020). For instance, separated transcription and translation processes were realized inside multicompartment droplets (Aufinger and Simmel, 2018). Artificial nuclei were encapsulated inside hydrogels through the water-in-oil-in-water double emulsion (Niederholtmeyer et al., 2018). The microfluidics could also provide an easily-operated platform for the induction of membrane-less compartments inside artificial cells (Deshpande et al., 2019a; Love et al., 2020). The technology can generate standard and monodisperse droplets as artificial cells, thus are widely-used and promising in this field, but they might suffer from poorly stable structures (Martino and deMello, 2016; Deshpande and Dekker, 2019).

Many enzymatic reactions mainly happen in cell-free solutions in artificial cells. However, the bio-membrane system is an important characteristic of life, which greatly increases the surface-to-volume proportion, supports functional proteins, and improves the efficiency of bioreactions. Inspired by this, the reconstitution of membrane proteins on synthetic membranes, such as lipids and amphiphilic block copolymers, has been broadly studied. These membrane proteins could form bio-pores to improve the mass transfer (Garni et al., 2017; Shen et al., 2018), induce the membrane deformation (Steinkuhler et al., 2020), or play a catalytic role (Choi et al., 2006). As artificial cells could provide a great platform for studying membrane proteins *in vitro*, more functional artificial membranes might be developed in the future. Compared with the complete synthesis, another appealing method is to utilize the natural membrane system. For example, thylakoid membranes were directly used to construct the artificial chloroplast and make outstanding achievements in optical conversion efficiency (Tarryn et al., 2020). These efforts have simplified the construction process while retaining the complexity of the artificial system (Figure 3, Structure hierarchy).

After the multicompartment of different functional modules, the question could be naturally put forward that whether these separated modules could function efficiently. Fortunately, the bioreactions such as protein synthesis had a better performance in a confined environment than in bulk solution (Park et al., 2009), probably due to the high local concentration of enzymes and substrates. However, it was not the case in an artificial cell with a higher structure hierarchy, for complex metabolism behaviors usually became difficult to reconstitute (Berhanu et al., 2019). This was partly attributed to low efficiency of the bioreactions *in vitro*, and continuous efforts to optimize the cell-free system would still make sense. In addition, the allocation of resources inside artificial cells was probably far away from the optimum. For example, in a passive diffusion case, the output of one module would diffuse outside and come into another module randomly. As a result, the signals might distribute evenly in the artificial cell, trigger the bioprocess in an inefficient way, and waste the limited resources in the confined space. To solve this problem, a distinctive and specific pathway is promoted for signal receiving and processing. For example, the complementary base pairing was applied in the intracellular communication of artificial organelles to trigger specific communication (Aufinger and Simmel, 2018). Afterward, the feedback system should be established, which means the different modules could regulate the production of signals based on the concentration to reach homeostasis. Through careful regulation, we might keep them at the optimum concentration to get the efficient allocation of materials and energy (Figure 3, Homeostasis). However, this would be pretty difficult as the precise operation of bioreactions inside a tiny compartment was still not so mature, and thus new technology is expected in this field.

The unity of different functional modules toward a complete artificial cell is a significant and laborious task, as the involved interactions are highly complicated. In this case, computer-aided technology would provide a more feasible and time-saving approach (Figure 3, Computer-aided). Computational and mathematical modeling has been widely used to investigate or predict the potential dynamics in artificial cells (Cho and Lu, 2020). These include the biochemistry effects in a confined environment (Sakamoto et al., 2018), the communication behaviors triggered by signal molecules (Rampioni et al., 2014), or the design of membrane proteins on lipids (Alford et al., 2020). Recently, the module assembling process was successfully established through the computer-aided design, which consisted of a membrane proliferating module, a membrane contraction module, and a positioning module (Schneider and Mangold, 2018). This result has greatly revealed the advance of computer-aided technology. It would help us to understand the complicated mechanism of synthetic bio-processes and bring new opportunities to the art of constructing artificial cells.

## 4 Challenges and Prospects

The construction of artificial cells is not a task that can be accomplished in the short term. It is an interdisciplinary field, which covers almost all of the scientific or engineering subjects. We proposed constructing artificial cells from functional modules to simplify the inherent complexity of this task. In this case, this complicated project could be separated into several smaller tasks, with the full use of strength worldwide. A bio-engineering workflow has been put forward to promote the standardization for the construction of artificial cells (Schwille et al., 2018). In the systematic design of a biosystem, four elements should be considered, including the precise definition of the biosystem’s functionalities, the experimental validation of the functional modules, the reliable assembling of different modules, and the production of the biosystem in a reproducible manner. If we consider an artificial cell a precise machine, we are still struggling to invent new accessories, assemble them and broaden the final functionalities.

### 4.1 Challenges

The functions of an artificial cell could be divided into four essential parts: metabolism, energy supplement, proliferation, and communication. Among these four parts, metabolism is the most fundamental, as the other three could be formally attributed to bioreactions. Up to now, only simple bioreactions have been realized when encapsulated inside artificial cells. The low efficiency and simplified reaction processes have greatly hindered the application of the synthetic compartments. In energy analysis, this could be partly attributed to the short supplement of energy. Therefore, more efficient energy supplements and their well-coupling with the core metabolic process are in urgent need. In the proliferation part, how to connect the replication of internal chemicals with the precise and reproducible division of compartments is the main challenge. As for the communication module, it is wondered how the communication among artificial cell populations could affect the development or differentiation of individuals. In other words, the interactions at a collective level are also crucial in describing an artificial cell. Furthermore, the integration of these different modules also requires more deep researches. The challenge would be the achievement of an elaborate organization rather than a simple stack. The latter might cause competition for resources and internal friction.

### 4.2 Possible Solutions

The challenges in the four modules and their efficient integration should be considered carefully, and several possible solutions are proposed here for further discussion. To improve metabolism efficiency and diversity, the cell-free system should be developed to support the robust synthesis of different proteins. The connotation of the system needs to be expanded. For example, the riboswitches, chaperons, or some post-translation modification factors could be introduced for regulation. The system could also perform not only as a synthesis machine but also as a rubbish factory to enable substrate circulation. At the same time, since the energy supply in a living cell is significant, the combination of natural energy parts and synthetic systems would be efficient as a transition toward a reconstituted energy supply system. The external supplement and self-regeneration could also coordinate in one single artificial cell to work more remarkably. As for the precise control of proliferation, the mechanical division through microfluidic technology might be a possible strategy. A self-driving division model might rely on the interaction between the replication of the interior and the membrane motility. For example, if the genetic materials were linked with the membrane, their replication might induce the change of the membrane morphology. Since there might be an optimized quantitative relationship between genes and membranes, precise and average division might be achieved. In the communication part, different types of releasing and receiving processes for signals might be developed except for different signals through one transmission mode. For example, the hybridization between antigens and antibodies could happen on the surface of artificial cells, while small molecules diffuse into the interior to function. In this case, a complex communication system could be established for further observation of collective behaviors. At last, for efficient integration of different modules, the multicompartment and membrane systems could be the structure basis, while the optimized allocation of resources and the feedback system toward homeostasis would finally support the well-organized bio-machine.

The modulization and unity strategy could be appropriate for the complicated subject of constructing artificial cells. The following work would concentrate on expanding the module library and the efficient assembling of these modules based on the desired function. Artificial cells are firstly mimics of living cells. Therefore, the bottom-up construction could probably gain inspiration from the natural world. However, these man-made bio-machines could be more than cells, which means that they could exhibit unprecedented phenotypes or have unordinary functions. With the development of relative fields, artificial cells could improve our cognition toward life and help to improve the quality of our real-life for their application in diagnostics and therapeutics.
